# Rethinking Disability

**DOI:** 10.1186/s12916-017-1002-6

**Published:** 2018-01-26

**Authors:** Alarcos Cieza, Carla Sabariego, Jerome Bickenbach, Somnath Chatterji

**Affiliations:** 1 Department of Management of Noncommunicable Diseases, Disability, Violence and Injury Prevention, World Health Organization, Geneva, Switzerland; 2 Department of Medical Informatics, Biometry and Epidemiology-IBE, Chair for Public Health and Health Services Research, Research Unit for Biopsychosocial Health, Ludwig-Maximilians-Universität (LMU), Munich, Germany; 3 Department of Health Sciences and Health Policy, University of Lucerne and Swiss Paraplegic Research (SPF), Nottwil, Switzerland; 4 Department of Information, Evidence and Research, World Health Organization, Geneva, Switzerland

**Keywords:** International Classification of Functioning, Disability and Health, Disability, Health Status Indicators, Model Disability Survey, Global Burden of Disease, Public Health

## Abstract

Disability as a health outcome deserves more attention than it has so far received. With people living longer and the epidemiological transition from infectious to noncommunicable diseases as the major cause of health burden, we need to focus attention on disability – the non-fatal impact of heath conditions – over and above our concern for causes of mortality.

With the first Global Burden of Disease study, WHO provided a metric that enabled the comparison of the impact of diseases, drawing on a model of disability that focused on decrements of health. This model has since been elaborated in the International Classification of Functioning, Disability and Health as being either a feature of the individual or arising out of the interaction between the individual’s health condition and contextual factors. The basis of WHO’s ongoing work is a set of principles: that disability is a universal human experience; that disability is not determined solely by the underlying health condition or predicated merely on the presence of specific health conditions; and finally, that disability lies on a continuum from no to complete disability. To determine whether interventions at individual or population levels are effective, an approach to disability measurement that allows for an appropriate and fair comparison across health conditions is needed. WHO has designed the Model Disability Survey (MDS) to collect information relevant to understand the lived experience of disability, including the person’s capacity to perform tasks actions in daily life, their actual performance, the barriers and facilitators in the environment they experience, and their health conditions. As disability gains prominence within the development agenda in the United Nations Sustainable Development Goals, and the implementation of the United Nations Convention on the Rights of Persons with Disabilities, the MDS will provide the data to monitor the progress of countries on meeting their obligations.

The lesson learned from WHO’s activities is that disability is a universal human experience, in the sense that everyone can be placed on a continuum of functioning and either currently experiences or is vulnerable to experiencing disability over the course of their lives. This understanding of disability is the key to mainstreaming disability within the public discourse.

## Background

Disability as a health outcome deserves more attention than it has so far received. We present here an argument for rethinking disability within clinical and public health contexts. There has been a recent global focus on disability as a development issue with a global impact [1]. With people living longer around the world and the epidemiological transition from infectious to noncommunicable diseases (NCDs) as the major cause of health burden, we need to focus attention on disability – the non-fatal impact of heath conditions – as well as causes of mortality. While it is important to know why people die, it is also important to understand how they live (with their health conditions). With population ageing and advances in medical technologies, people everywhere are living with multiple chronic conditions and experience higher levels of disability. An integrated approach to healthcare requires a focus on improving health and reducing disability and not merely controlling disease symptoms.

To determine whether health interventions at the individual or population levels are producing the desired health gains, we need a clear conceptualization of non-fatal health outcomes and an approach to measurement of disability that allows for an appropriate and fair comparison of the impact of different health conditions (disorders, diseases, and injuries) as well as the gains from clinical and public health interventions. We need to quantify the magnitude of these health gains over time at the individual level, which can then be aggregated over the population rather than simply dividing the population of interest into those who are disabled and those are not. Although disability advocates will require estimates of prevalence of disability in order to argue for the right policy, we should take care how these estimates are created and what they will be used for. Rather than merely counting the number of people, for example, with chronic health diseases as a proxy measure of disability in the population, it would be far preferable to actually determine the extent of disability across the population irrespective of the underlying health condition, and use some plausible threshold, as fit for purpose, to determine population prevalence. Quantifying the magnitude, in short, requires a measure that is continuous, that is, a measure of an amount rather than a count.

## Main text

The World Health Organization (WHO) has systematically developed a common conceptualization and an approach to measurement of disability. With the first Global Burden of Disease (GBD) study [2], WHO provided a metric that enabled the comparison of the impact of diseases. This allowed, for example, a comparison of diabetes with depression and provided a measure of parity between physical and mental disorders. The GBD study drew on a model of disability that focused on decrements of health. This model has since been elaborated in the *International Classification of Functioning, Disability and Health* (ICF) as being either an intrinsic feature of the individual or arising out of the interaction between the individual’s health condition and contextual factors [3]*.* This conceptualisation continues to be reflected in WHO’s World Report on Disability [4] and in WHO’s World Report on Ageing and Health [5].

Three consensus points about health formed the basic components of the face validity of WHO’s health measurement strategy: that health is a determinant of, but does not coincide with wellbeing; that health is a function of states or conditions of the human body or mind, constituted by the person’s intrinsic capacity to execute specific tasks and actions in a range of domains that capture the full breadth of human functioning; and that health is an intrinsic feature of the individual [6]. Taken together, health for measurement purposes is conceptualized as an aggregate across domains of functioning that are intrinsic to the person and describes the health status of an individual. This can be further aggregated over the population.

It follows that human functioning, and decrements of functioning or disability, lies on a continuum. While individuals may have very different profiles of functioning across different domains characteristic of their underlying health conditions, these can be combined such that the extent of functioning (or disability) can be compared across these different profiles. Whenever decrements in health are being quantified, either by means of data from population health surveys or in the estimates underpinning global health reporting, a common conceptual basis allows them to be compared along a single scale.

The basis of all of WHO’s ongoing work is a set of principles: The first is that disability is a universal human experience, not a mark of a demographic minority, although given the social stigma often associated with disability, it is understandable if advocates insist that theirs constitutes a minority identity. Secondly, disability is etiologically neutral, in the sense that this decrement in functioning is not linked to, or solely predicated on the presence of a specific health condition characterized by signs and symptoms. Having difficulties leaving one’s home, whether it stems from restricted mobility due to spinal cord injury or a pathological fear of open spaces as in agoraphobia, has comparable impact on a person’s life. Etiological neutrality ensures a parity between disability arising from physical and mental health conditions. Lastly, disability lies on a continuum from no disability (full functioning) to complete disability. This continuum can be partitioned by a threshold identified as fit for purpose, including, for example, advocacy for policy change. Because disability is a continuous phenomenon, changes in this quantity can be tracked over time, and across individuals and populations, including, in particular, changes linked to clinical or public health interventions. Because disability is continuous, it is also universal, since over the course of a person’s life the chances are extremely high that, in some domain, he or she will experience some decrement in functioning. In other words, human functioning ranges from full functioning to some limitation in functioning to complete loss in functioning.

Unfortunately, information on disability is often used only to dichotomize the population into those who are ‘disabled’ and those who are not without capturing the entire breadth of the disability spectrum. This approach typically entails counting impairments, such as blindness, deafness and intellectual impairment, or counting limitations in some specific domains of functioning or in Activities of Daily Living (ADLs) and Instrumental Activities of Daily Living (IADLs). Counting impairments, or limitations in functioning in individual domains, ADLs and IADLs does not allow one to quantify the magnitude of impact of different health conditions in a comparable manner. Yet a majority of people who currently receive disability benefits and supports are those with chronic diseases accompanied by a significant level of disability, rather than specific impairments [7]. Furthermore, by not quantifying the magnitude of disability in a comparable manner across these health conditions there is no way of determining whether any particular individual has more or less disability than another, and so is in need of more or fewer resources. Although ADLs and IADLs are etiologically neutral, by their very nature of being designed to capture disability in older adults, they fail to capture mild and moderate decrements in health along the entire spectrum of functioning over the life-course, inasmuch as a person will only experience these difficulties if their disability has crossed a certain threshold. ADLs refer to the basic tasks of everyday life, such as eating, bathing, dressing, toileting, and transferring and inability to execute these tasks indicate some degree of dependence and the need for assistance [8, 9]. It is important to be able to identify people with milder limitations, since these can be the focus of public health interventions and are likely to produce the most gain in population health.

WHO has long recognized that the lived experience of disability is significantly influenced by people’s real-life environments. Interventions to make the environment more facilitating enable people to do the things that matter to them and that improve wellbeing. Health and social interventions need to be tailored to improving health status, making the environment more facilitating through the provision of assistive technology and personal assistance, and making the built, attitudinal and social environments more accommodating, accessible and conducive to better functioning. Clearly, it is also crucial that we have the ability to determine, quantitatively, which of these interventions are the most effective and the most cost-effective. Adequate, effective and reasonable interventions in all three domains can only be accomplished if we have a complete understanding of the experience of disability operationalized for measurement purposes.

Every clinician should appreciate that his or her patient experiences their health in terms of how it impacts their daily life, fully contextualized by their environment. Diagnosis of signs and symptoms is an essential tool, but from the patient’s perspective what matters is what she or he can or cannot do in daily life. This is all the more relevant where the presence of comorbidity, very common in NCDs, means that managing individual diseases is often unlikely to produce the desired outcomes. Clinicians, or others, called upon to apply a protocol for determining disability for social supports or services need to realize that the impact of the person’s actual environment can – positively or negatively – make all the difference. A person with a severe health condition may, with supports, be able to fully participate in school, family and work; while a person with several mild health conditions, in an unaccommodating environment, may experience near total disability in these same domains.

Having laid the groundwork for this understanding of disability, WHO has designed and implemented the Model Disability Survey (see Table 1) that collects information relevant to the intrinsic capacity of individuals to quantify levels of health, the actual performance of tasks and actions in day-to-day life, and barriers and facilitators in the environment in order to understand the lived experience of disability [10]. As disability gains prominence within the development agenda in the United Nations Sustainable Development Goals [1], and the human rights agenda with the implementation of the United Nations *Convention on the Rights of Persons with Disabilities* [11]*,* the MDS provides countries with the needed data to monitor progress towards achieving their obligations. The MDS effort is, moreover, linked to Universal Health Coverage and other prominent WHO activities, such as the global strategies and action plans for disability (WHA66.9) [12], mental health (WHA66.8) [13], noncommunicable diseases (EB130.R7) [14] and ageing (A69/17) [15]. The MDS approach not only makes it possible to disaggregate outcomes of interest along the disability continuum for those who experience severe, moderate and mild disability, as identified by suitable thresholds. But the MDS also enables decision-makers to go beyond to identifying the factors that are responsible for inequalities allows them to identify appropriate and effective interventions and policies.

**Table 1:** Characteristics of both versions of the Model Disability Survey

	MDS Stand-alone Version	MDS Brief version
Goal	National or regional implementations as a dedicated standalone disability survey	Integration in existing household surveys as a disability module
Implementation	every 5 to 10 years	flexible, continuous
Developed in	2012	2016
Length in time	60-120 minutes	10-15 minutes
Core modules:		
Environmental factors	This module contains a broad inventory of questions about: - Hindering or facilitating aspects of the general environment - Use and need for personal assistance - Family and social support - Attitudes of others - Accessibility to information - Regular use of medication - Use and need for assistive devices for self-care, mobility, cognition, seeing and hearing - Presence and need of modifications at home, school, work and community.	Contains 13 questions about: - Hindering or facilitating aspects of the general environment - Use and need for personal assistance - Family and social support - Attitudes of others - Use and need for assistive devices.
Functioning	The module includes 47 questions covering the actual performance of tasks and actions in day-to-day life in the following 17 functioning domains: mobility, hand and arm use, self-care, seeing, hearing, pain, sleep and energy, breathing, affect, interpersonal relationships, handling stress, communication, cognition, household tasks, community and citizenship participation, caring for others and work and schooling. All questions target performance considering health conditions and the physical, social, attitudinal and political environment of the person.	Contain 12 questions of the standalone version, selected for their ability to generate individual disability scores comparable to the ones generated with the standalone version.
Health conditions and capacity	Altogether 17 questions target the intrinsic capacity of a person, determined solely by health conditions, in the same 17 domains covered in the functioning module. Additionally, a self-report part about the presence of health conditions and impairments is included. If a respondent endorses a health condition, three questions follow: 1) whether any health professional has ever diagnosed it; 2) whether the person has been given any medication in the last 12 months; and 3) whether the person has been given any other kind of treatment, beyond medicines, in the last 12 months.	Contain 12 questions of the standalone version, selected for their ability to generate individual capacity scores comparable to the ones generated with the standalone version.

## Conclusions

The important lesson learned from WHO’s activities conceptualizing and measuring disability is that disability is a universal human experience, in the sense that everyone can be placed on a continuum of functioning and either currently experiences or is vulnerable to experiencing disability over the course of their lives. This understanding of disability is the key to mainstreaming disability within the public discourse. Truly, disability is about all of us, and as disability advocates rightly say, disability must be mainstreamed in society and throughout health and social policy such that it indeed becomes everyone’s business.

## Abbreviations

ADLs: Activities of Daily Living
GBD: Global Burden of Disease
IADLs: Instrumental Activities of Daily Living
ICF: International Classification of Functioning, Disability and Health
MDS: Model Disability Survey
NCDs: Noncommunicable diseases
WHO: World Health Organization
